# Drug-induced movement disorder: A disproportionality analysis using the FDA adverse event reporting system (FAERS) from 2004 to 2024

**DOI:** 10.1371/journal.pone.0335449

**Published:** 2025-10-31

**Authors:** Jingjing Zou, Chenju Zhan, Yan Feng, Qixi Liu, Guohao Peng, Zhongjie Cai, Lingling Luo, Jun Zou

**Affiliations:** 1 Department of Nursing, Mindong Hospital Affiliated to Fujian Medical University, Ningde, China; 2 Department of Endocrinology, Mindong Hospital Affiliated to Fujian Medical University, Ningde, China; 3 Department of Pharmacy, Mindong Hospital Affiliated to Fujian Medical University, Ningde, China; The University of Texas Rio Grande Valley School of Medicine, UNITED STATES OF AMERICA

## Abstract

**Background:**

Although many drugs have been associated with drug-induced movement disorders (DIMDs), the associated risks are unclear. This study aimed to identify high-risk drugs for DIMDs through disproportionality analysis of the Food and Drug Administration Adverse Event Reporting System (FAERS) database and to explore risk factors for DIMDs through sensitivity analysis.

**Methods:**

Four disproportionality analysis methods were used to assess the risk signals of drugs that may induce DIMDs from the first quarter of 2004 to the fourth quarter of 2024. One-way analyses, LASSO analyses, and logistic regression analyses were performed to explore the risk factors associated with DIMDs.

**Results:**

There are 138,081 reports related to DIMDs. This study identified 148 suspected drugs. Age under 33 years, male gender, and 62 medications, including METOCLOPRAMIDE, ARIPIPRAZOLE, CARBIDOPA, LEVODOPA, RISPERIDONE, and QUETIAPINE, are all independent risk factors for drug-induced movement disorders. The area under the ROC curve (AUC) reflecting model predictive accuracy was 0.724.

**Conclusion:**

Our disproportionality analysis and sensitivity analysis of the FAERS database identified drugs potentially associated with DIMDs. These findings can provide valuable information for clinicians to be more cautious when prescribing these drugs and to monitor patients for the development of movement disorders closely. Additionally, the results can help regulatory agencies make informed decisions regarding the safety of drugs.

## Introduction

Drug-induced Movement Disorder (DIMDs) is a type of adverse reaction caused by drug treatment. It not only seriously affects the quality of life of patients but also may lead to a decrease in their compliance with treatment, and even cause other complications, posing a significant challenge to their health [1]. It covers various abnormal motor manifestations, such as Parkinson's syndrome, dystonia, chorea, tremor, etc [2,3], and have a complex mechanism of onset that is mainly related to imbalances in the dopaminergic system, abnormalities in the gamma-amino butyric acid (GABA) ergic system, oxidative stress and mitochondrial dysfunction, and the interplay of neurotransmitters and neuromodulators [4]. Over the past few decades, with the continuous increase in the types of drugs and the increasingly wide clinical application, the cases of drug-induced movement disorders have gradually increased, many studies have shown that DIMDs can be triggered by a variety of drugs, such as antipsychotics [5], gastric stimulants [6], antidepressants [7], antiepileptics [8], and calcium channel blockers [9].

There are still a large number of medications for which the potential association with DIMD remains unclear. This makes it difficult for clinicians to accurately assess the risk of drug-induced movement disorders during medication administration, which may prevent timely and effective prevention and intervention. Healthcare professionals need to be able to recognise DIMDs at an early stage and manage them correctly. However, the true prevalence and incidence of DIMDs are often underestimated. This is due in part to underreporting by healthcare professionals and patients, as well as the difficulty in accurately diagnosing these disorders, which usually exhibit milder or variable symptoms [10].

Pharmacovigilance, a science and activity related to the detection, assessment, and prevention of adverse drug reactions or any other drug-related problems, plays a critical role in monitoring DIMDs [11]. The U.S. Food and Drug Administration Adverse Event Reporting System (FAERS) database is a valuable resource for pharmacovigilance research [12]. The database contains a wealth of real-world adverse drug event data reported by U.S. healthcare professionals, consumers, and pharmaceutical manufacturers [13]. By analyzing the FAERS database, researchers can identify potential associations between drugs and DIMDs, discover new or rare cases, and develop hypotheses for further research [14].

Disproportionality analysis is a statistical method that utilises large-scale adverse reaction reporting data to determine whether there is an unusual association between a drug and an adverse reaction. It compares the difference between the actual frequency of reporting and the expected frequency of reporting an adverse reaction to a particular drug [15]. This method can quickly screen potentially risky drugs from a large dataset, thereby filling a gap in existing research and providing valuable insights for further research and informed regulatory decision-making. In this study, we aimed to conduct a real-world pharmacovigilance analysis using the FAERS database to explore the epidemiologic profile of DIMDs, identify the medications most commonly associated with these disorders, and assess the risk factors and patient characteristics related to DIMDs. Our results may contribute to a better understanding of DIMDs in real-world settings and provide valuable information to improve patient care and medication safety.

## Methods

### Data sources and collection

This retrospective pharmacovigilance study analysed the FAERS database using disproportionality analysis. Since 2004, FAERS has been providing quarterly updates of AERs based on the International Conference on Harmonisation (ICH) E2B guidelines to support post-marketing surveillance of various drugs and biologics [16].

Therefore, we collected data on DIMDs from FAERS from the first quarter of 2004 to the fourth quarter of 2024. Each file contains seven types of data, including DEMO (containing patient demographic and administrative information, a single record for each event report), REAC (containing all Medical Dictionary of Regulatory Activities (MedDRA) terms coded for the event), DRUG (containing the same amount of drug/biological information as the event report), OUTC (containing patient outcomes for the event), RPSR (containing the event report), THER (contains the medication start date and end date of the reported medication) and INDI (contains all MedDRA terms coded for the use/diagnostic indication of the reported medication). Duplicate data were removed as recommended by the FDA. If cases had the same case ID, the most recent statement with the fda_dt was retained. If the case ID and fda_dt were the same, the statement with the larger master ID was retained. After deduplication, some primary ID duplicate entries were still found, so secondary deduplication was performed to resolve these remaining duplicates. The reporting in this study followed the PharmacoVigilance (READUS-PV) guidelines for disproportionality analyses in drug safety signal detection, using individual case safety reports [17].

Adverse events (AEs) in the REAC are coded by Preferred Terminology (PT) according to MedDRA. DIMDs include MUSCLETWITCHING, TIC, TARDIVEDYSKINESIA, DROOLING, DYSKINESIA, CHOREOATHETOSIS, MOVEMENTDISORDER, GRIMACING, MOTORDYSFUNCTION, EXTRAPYRAMIDALDISORDER, DYSKINESIANEONATAL, CHOREA, BUCCOGLOSSALSYNDROME, ATHETOSIS, BALLISMUS, DYSKINESIAOESOPHAGEAL, RESPIRATORYDYSKINESIA, OCULOGYRICCRISIS, DOPAMINEDYSREGULATIONSYNDROME, PROTRUSIONTONGUE, RABBITSYNDROME, PHARYNGEALDYSKINESIA, ABNORMALINVOLUNTARYMOVEMENTSCALE, CHRONICTICDISORDER, SECONDARYTIC, PROVISIONALTICDISORDER, OCULOGYRATION, REDUCEDDEXTERITY. Drugs associated with AEs were assigned different roles (prime suspect, secondary suspect, concomitant, interaction), and cases were included only if the drug was classified as a "prime suspect" for DIMDs.

### Statistical analysis

Descriptive analyses were used to summarise the clinical characteristics of DIMDs, including the number of reported cases, gender, age, weight, identity of the reporter, country or region of reporting, and clinical outcome indicators. Individuals aged 120 years or older and weighing 400 kg or more were defined as outliers and excluded from the analysis.

### Disproportionality analysis

In this study, we constructed a 2 × 2 list of columns (S1 Table), and the disproportionality analyses used included the Reporting Odds Ratio (ROR), Proportional Reporting Ratio (PRR), Bayesian Confidence Propagation Neural Network (BCPNN), and Empirical Bayes Geometric Mean (EBGM). The relationship between DIMDs and suspected drugs was assessed using disproportionality analysis, and signals were considered valid only if their values met the thresholds of all four algorithms and their reporting frequency was at least 3 (S2 Table). Positive signals satisfying all four algorithms were further highlighted in terms of strength of association between suspected drugs and DIMDs in the form of volcano plots (-log(p-adjust) as the horizontal coordinate and logROR as the vertical coordinate) represented by the ROR and p-adjust (Fisher's exact test and Bonferroni-corrected p-value).

### Sensitivity analysis

To further analyse the positive signals and verify their stability, we extracted FAERS reports containing patient information (gender, age), and only reports with complete data were analysed. One-way analyses of suspected drugs were performed using the ROR and corresponding lower bounds of the 95% CI of >1, a > 100, and p-adjust<0.01, and least absolute shrinkage and selection operator (LASSO) regressions were performed. Multifactorial logistic regression and the Bonferroni correction were used to identify independent risk factors for DIMDS, with LASSO-screened medications and basic patient information serving as the dependent variables. To select the most predictive subset of suspected drugs and covariates, we performed a least absolute shrinkage and selection operator (LASSO) logistic regression with 10-fold cross-validation. All 108 candidate binary variables were entered simultaneously: demographic indicators (male sex, age < 33 years); the 62 suspected drugs that met the univariate screening criteria (report count > 100, ROR lower 95% CI > 1, Bonferroni-adjusted p < 0.01); seven comorbidity flags derived from MedDRA preferred terms (Parkinson's disease, schizophrenia, affective disorders, epilepsy, gastro-oesophageal reflux, nausea/vomiting, and other CNS disorders). The glmnet package (v4.1-8) in R 4.4.3 was used with α = 1 (pure Lasso), standardisation = TRUE, convergence threshold = 1 × 10 ⁻ ⁶, and maximum iterations = 100 000. Cross-validation (10-fold, stratified) identified the optimal λ by the minimum mean-squared prediction error (λ.min). The final model retained variables with non-zero coefficients: male sex, age < 33 years, and 13 individual drugs (metoclopramide, aripiprazole, carbidopa-levodopa, risperidone, quetiapine, valbenazine, olanzapine, pregabalin, paliperidone, haloperidol, ciprofloxacin, levofloxacin, and imipenem). These variables were then entered into the multivariable logistic regression with Bonferroni correction.

All analyses were performed using R software (version 4.4.3).

### Study design

**Fig 1:**
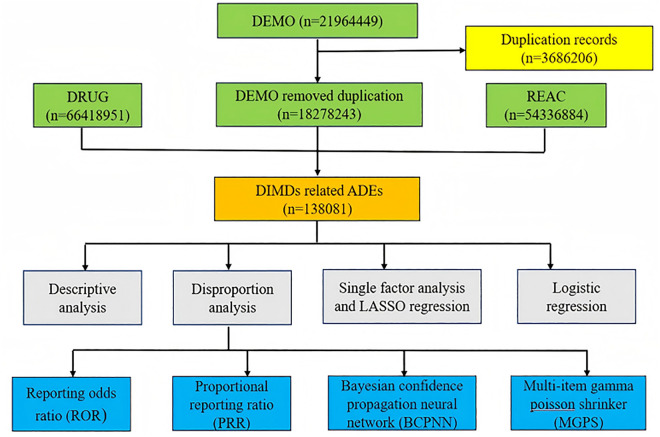
The overall study design.

The overall study design is illustrated in Fig 1.

## Results

### Baseline characteristics of DIMDs

**Fig 2:**
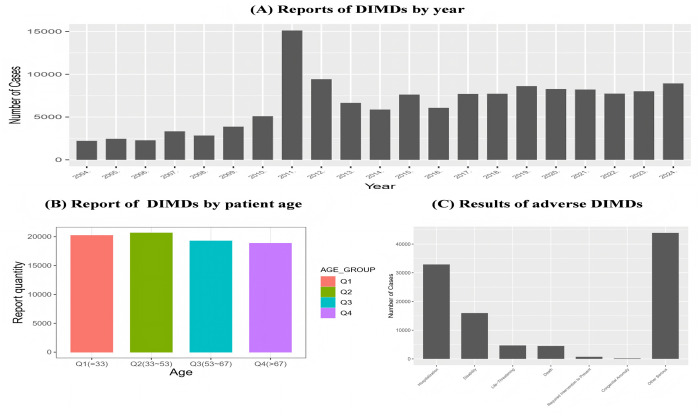
Information related to DIMDs.

From the first quarter of 2004 to the fourth quarter of 2024, FAERS recorded 138,081 reports related to DIMDs, with the yearly distribution of these reports shown in Fig 2(A). This distribution exhibits an overall upward trend over the period, with the highest number of reports recorded in 2011.

Baseline characteristics of DIMDs (as shown in Table 1). There were 75,188 (54.45%) reports of females, 49,647 (35.95%) reports of males, and 13,246 (9.60%) reports of missing gender values. The median age of the patients was 53 years (interquartile range [IQR], 33–67 years), and the median weight was 70.0 kg (IQR, 57.2–85.0 kg). The most frequently reported occupation was consumer (55,442 cases; 40.15%). Among the reporting countries, the United States, the United Kingdom, and Canada reported the highest numbers of cases of DIMDs, at 85,828 (62.16%), 8,559 (6.20%), and 6,054 (4.38%), respectively.

**Table 1:** Baseline characteristics of DIMDs.

Characteristics	Drug-induced movement disorders (N = 138081)
**Age**	
Median (Q1, Q3)	53.0 (33.0,67.0)
Unknown	59712 (43.24%)
**Weight**	
Median (Q1, Q3)	70.0 (57.2,85.0)
Unknown	101772 (73.70%)
**Gender**	
Female	75188 (54.45%)
Male	49647 (35.95%)
Unknown	13246 (9.60%)
**Occupation of the reporter**	
Consumer	55442 (40.15%)
Physician	25155 (18.22%)
Lawyer	18831 (13.64%)
Other health-professional	14676 (10.63%)
Health-professional	10332 (7.48%)
Pharmacist	5878 (4.26%)
Registered nurse	62 (0.04%)
Unknown	7705 (5.58%)
**Country of the reporter**	
United States	85828 (62.16%)
United Kingdom	8559 (6.20%)
Canada	6054 (4.38%)
France	4555 (3.31%)
Germany	3358 (2.43%)
Japan	3017 (2.18%)
Italy	1831 (1.33%)
Netherlands	1673 (1.21%)
Brazil	1572 (1.14%)
China	1427 (1.03%)
Other	20207 (14.63%)

The study results indicated that DIMDs had the highest number of reported cases in patients aged 33–53 years, followed by those aged 33 years and younger. Fig 2(B). Among the adverse effect outcomes, the most common adverse outcome was prolonged hospitalisation, followed by patient disability, as shown in Fig 2. (C).

### Drugs associated with DIMDs

Through the mutual validation of four disproportionality analysis methods, we identified a total of 148 high-signal-intensity drugs associated with DIMDs. These drugs included METOCLOPRAMIDE (ROR = 766.5, PRR = 129.19, EBGM = 112.23, IC = 6.81), ARIPIPRAZOLE (ROR = 12.89, PRR = 111.88, EBGM = 11.42, IC = 3.51), CARBIDOPA. LEVODOPA (ROR = 15.92, PRR = 14.39, EBGM = 13.86, IC = 3.79), RISPERIDONE (ROR = 9.19, PRR = 8.68, EBGM = 8.42, IC = 3.07), QUETIAPINE (ROR = 6.15, PRR = 5.93, and EBGM = 5.81, IC = 2.54), VALBENAZINE (ROR = 20.41, PRR = 17.88, EBGM = 17.52, IC = 4.13), OLANZAPINE (ROR = 6.71, PRR = 6.44, EBGM = 6.35, IC = 2.67), PREGABALIN (ROR = 2.39, PRR = 2.37, EBGM = 2.35, IC = 1.23), PALIPERIDONE (ROR = 6.9, PRR = 6.61, EBGM = 6.54, IC = 2.71), HALOPERIDOL (ROR = 23.04, PRR = 19.84, EBGM = 19.6, IC = 4.29) and others (S3 Table).

**Fig 3:**
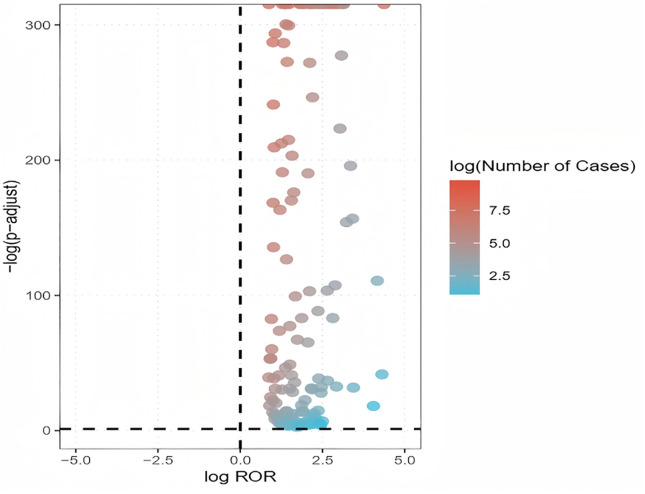
Volcano map of suspected drugs associated with DIMDs.

We further analysed the suspected drugs associated with DIMDs in the form of volcano plots (Fig 3), demonstrating the strength of the alleged drugs in terms of the intensity of their association with DIMDs. In the Fig 3, the x-axis represents the logarithm of the ROR. The positive x-axis indicates that adverse reactions associated with DIMDs were reported more frequently than other adverse reactions. The y-axis indicates the negative logarithm of the p-value after Fisher's exact test and Bonferroni correction. The positive y-axis shows highly significant differences. The colour of the dots indicates the logarithm of the number of case reports. The redder the colour, the higher the number of reports. Thus, the graph shows that all these 148 suspected drugs associated with DIMDs have significant differences and signal strengths.

### Sensitivity analysis of risk factors for DIMDs

**Fig 4:**
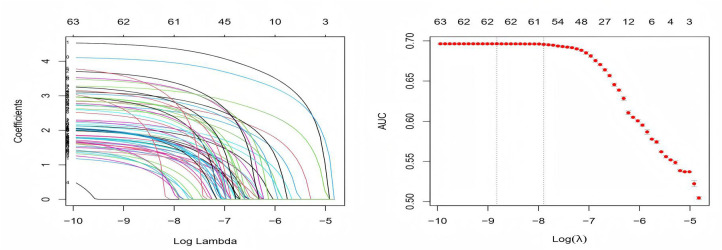
Cross-validation results graphs.

**Fig 5:**
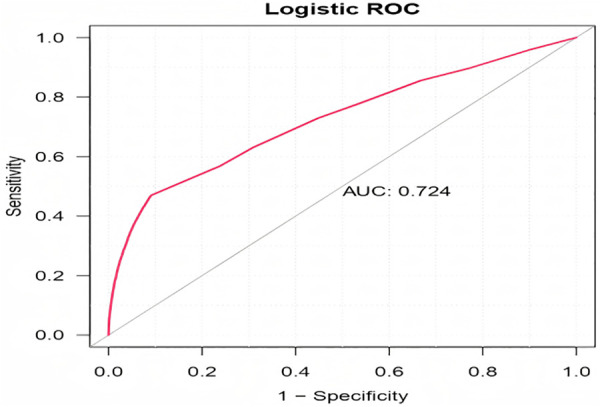
ROC curves for risk factors for DIMDs.

Suspected drugs with >100 case reports, lower limit of ROR (95% CI) >1, and *p*-adjust < 0.01 were extracted for one-way analysis and LASSO regression, and a total of 62 drugs were screened (Fig 4). Multifactorial logistic regression analysis was performed in combination with patient information (S4 Table); Age under 33 years (*p* < 0.01), male gender (*p* < 0.05), and METOCLOPRAMIDE, ARIPIPRAZOLE, CARBIDOPA, LEVODOPA, RISPERIDONE, QUETIAPINE, and 62 other drugs were independent risk factors for drug-induced dyskinesia. The ROC-AUC, indicating the predictive accuracy of the model, was 0.724 (Fig 5).

## Discussion

In this study, DIMDs were comprehensively analysed based on the U.S. Food and Drug Administration (FDA) Adverse Event Reporting System (FAERS) data from 2004 to 2024. The study identified a total of 148 drugs associated with DIMDs by four disproportionality analysis methods. The synergistic use of these algorithms effectively reduces the false positives and improves the accuracy of detecting rare adverse events. The results of multifactorial logistic regression showed that being male, under 33 years old, and using 62 specific drugs, including METOCLOPRAMIDE, ARIPIPRAZOLE, CARBIDOPA, LEVODOPA, RISPERIDONE, and QUETIAPINE, were identified as risk factors for drug-induced movement disorders (DIMDs). The results of this study not only further analysed and investigated the 148 positive drugs screened, but also verified the stability of the disproportionality analysis methods used in this study and the accuracy of the results.

From the first quarter of 2004 through the fourth quarter of 2024, there has been an overall upward trend in reports related to drug-induced movement disorders (DIMDs) in FAERS. On the one hand, healthcare system monitoring and reporting mechanisms for adverse drug reactions have improved over time, allowing more cases of DIMDs to be detected and reported on time. On the other hand, the types and frequency of drug use are increasing, and new drugs continue to enter the market, which may also lead to an increase in the number of DIMDs cases [18]. Among the reporting countries, the United States, the United Kingdom, and Canada reported the highest number of cases, which may be related to the countries’ large population bases, well-developed healthcare systems, and well-established systems for monitoring adverse drug reactions [19]. The most common adverse outcome is prolonged hospitalization [20], which not only increases the patient's healthcare costs but also imposes a financial burden on the patient and their family, and has a profound impact on the patient's health and quality of life. The second is the resultant disability, [21] which not only reduces the patient's self-identity and self-esteem but also causes the patient to lose their independence and autonomy in life, resulting in far-reaching negative impacts on their future life and further aggravating the patient's suffering and social burden. Therefore, clinicians should be more cautious when using drugs that may lead to DIMDs, pay close attention to patient's responses, and detect and manage adverse reactions on time [20].

In terms of age-related frequency, the highest proportion of people was found in the age group under 53 years. Additionally, the multifactorial analysis indicated that being under 33 years old is an independent risk factor for DIMDs. It is possible that the younger population is an essential factor in the occurrence of DIMDs[1]. The nervous system of this population may be undergoing dynamic changes, becoming more sensitive and active. Consequently, the interaction between drugs and neurotransmitter systems is more likely to trigger abnormal nerve signalling at this stage, leading to movement disorders. On the other hand, unhealthy lifestyles, such as staying up late, smoking, and alcohol abuse, may also adversely affect the nervous system and increase the risk of developing DIMDs[21]. Additionally, multifactorial analysis has demonstrated that males are an independent risk factor for DIMDs. Androgens are found at higher levels in men [22], and they may affect the stability of the neurotransmitter system and the function of nerve cells [23]. It has been shown that androgens can modulate the expression and function of dopamine receptors [24,25], thereby increasing the risk of DIMDs in men. In addition, men usually have more smoking and alcohol abuse habits. Long-term smoking can lead to cerebral vasoconstriction, affecting the blood supply to the brain and decreasing the stability of the nervous system. At the same time, alcohol abuse may directly damage nerve cells and disrupt the neurotransmitter balance [26]. The synergistic effect of these poor lifestyles and medications may further increase men's risk of developing DIMDs.

The high signal intensity drugs identified in this study by the four disproportionality analysis methods involved various categories such as antipsychotics (32), antiparkinsonian drugs (22), antidepressants (18), antiepileptics (13), and gastrointestinal dynamics (6). The signal strength associated with the gastrointestinal power drug METOCLOPRAMIDE was extremely high (ROR = 766.5, PRR = 129.19, EBGM = 112.23, IC = 6.81). When combined with the multifactorial analysis, metoclopramide was found to be an independent risk factor with OR (95% CI) = 103.5965 (91.8544, 116.6949). The signal strength exhibited by metoclopramide suggests that it has a significant impact on DIMDs. Metoclopramide is a dopamine D₂ receptor antagonist that blocks dopamine D₂ receptors in the central nervous system [27], particularly in the medullary emetic chemoreceptor zone (CTZ), and the gastrointestinal tract, and at therapeutic doses it exerts its antiemetic effect by blocking D₂ receptors in the CTZ [28], however, several studies have demonstrated a positive correlation between the development of DIMDs and the dose of the drug, and that when used at prolonged or high doses it excessively blocks dopamine D₂ receptors in the basal ganglia, disrupting the balance between dopaminergic and cholinergic neurotransmitter systems in the basal ganglia and leading to an extrapyramidal response [29,30]. This may be an essential pharmacological mechanism underlying the high signalling intensity of metoclopramide. In addition to dopamine receptors, metoclopramide may have effects on the serotonin (5-hydroxytryptamine, 5-HT) system [31], which also plays an essential role in the regulation of mood, sleep, and motor function, and may indirectly interfere with the normal functioning of dopaminergic neural pathways by affecting the release, uptake, or receptor binding of serotonin, increasing the risk of delayed DIMDs. Furthermore, GABA is the primary inhibitory neurotransmitter in the central nervous system and plays an essential role in the stabilisation of motor function [32]. The blocking effect of metoclopramide on dopamine receptors results in the weakening of GABAergic inhibition, thereby increasing the excitability of motor neurons and triggering involuntary movements. This may be one of the reasons why it leads to high signal intensity in DIMDs. Metoclopramide is a commonly used antiemetic drug, which is widely used in clinical practice and is used relatively frequently due to its many indications, such as nausea, vomiting, and dyspepsia caused by various reasons. Frequent use increases the cumulative exposure of the drug in the body, leaving dopamine receptors in a blocked state for a prolonged period of time, thereby increasing the risk of DIMDs, which further increases the likelihood of its occurrence in DIMDs, which in turn leads to the demonstration of high signal intensity in signal detection.

Among the 148 drugs identified, antipsychotics are the most numerous, and such drugs are the first-line medications for the treatment of psychiatric disorders, as well as the common drugs that trigger DIMDs [33]. Most antipsychotic drugs exert their therapeutic effects mainly by blocking the dopamine D2 receptor; however, long-term use can lead to dopamine receptor hypersensitivity, and once the blocking effect of the drug on the receptor is weakened or lifted, the hypersensitive dopamine receptor becomes overexcited, leading to involuntary movements and triggering DIMDs [34]. Aripiprazole, risperidone, quetiapine, olanzapine, and paliperidone were among the identified high signal intensity antipsychotics, highly consistent with the results of the multifactorial analysis. Combined with the multifactorial analysis, the independent risk factor aripiprazole had an OR (95% CI) = 18.0311 (16.8339,19.2931), which is a partial agonist at the dopamine D2 receptor, and also affects the 5-hydroxytryptamine receptor, which exerts its therapeutic effect by modulating the neurotransmitter systems such as dopamine and 5-hydroxytryptamine when treating psychiatric disorders [35], however, the over modulation of the dopamine system may affect the function of the basal ganglia, which plays a key role in motor control, and its dysfunction can lead to involuntary movements, which in turn can trigger DIMDs. Drugs such as risperidone are similar in that they have different affinities for dopamine and 5-hydroxytryptamine receptors, and vary in the degree of balance of neurotransmitters affected, which may explain the differences in signalling intensity.

Antiparkinsonian drugs were also the main drugs identified as causing DIMDs. The higher signal CARBIDOPA.LEVODOPA (ROR = 15.92, PRR = 14.39, EBGM = 13.86, IC = 3.79) also acted as an independent risk factor in the results of multifactorial analyses (OR (95% CI) =36.9697(34.6073,39.4608)). Carbidopa-levodopa is a classic drug combination for the treatment of Parkinson's disease [36]. Levodopa crosses the blood-brain barrier and is converted to dopamine in the brain, supplementing the lack of dopamine in the brain of patients with Parkinson's disease. In contrast, carbidopa inhibits the activity of peripheral dopa decarboxylase, reducing levodopa decarboxylation in the periphery and allowing more levodopa to enter the brain, thereby enhancing its therapeutic effect [37]. However, long-term use of carbidopa-levodopa can lead to abnormal fluctuations in dopamine levels in the brain, and such fluctuations can lead to changes in the sensitivity of dopamine receptors, an imbalance in the up- and down-regulation processes of receptors, and disruptions in the functioning and regulatory mechanisms of dopamine receptors, increasing the risk of DIMDs. Many clinical studies have followed patients on long-term use of carbidopa-levodopa and found that the incidence of DIMDs gradually increased with the prolongation of the drug's duration, suggesting a significant time-effect relationship between carbidopa-levodopa and DIMDs, further confirming the correlation between the drug and DIMDs [1]. The multifactorial analysis comprehensively considered a variety of factors that may affect the occurrence of DIMDs. It excluded the interference of other confounding factors, further clarifying the causal relationship between carbidopa-levodopa and DIMDs, which is also an essential piece of evidence supporting its high correlation signal strength.

Notably, among the 62 drugs in the results of the multifactorial logistic regression analysis, the identification of antibiotics, including ciprofloxacin OR (95% Cl) = 10.354 (9.5731,11.1805), levofloxacin OR (95% Cl) = 7.7752 (7.0627,8.5374), and imipenem OR (95% Cl) = 10.0825 (8.032,12.4753), was observed for antibiotics. The mechanism of such DIMDs is unclear, and the results of the current relevant potential studies suggest that levofloxacin, imipenem, and ciprofloxacin may affect the activity of calcium channels, leading to abnormally high intracellular calcium concentrations, which involves the release of neurotransmitters and signalling [38,39], and consequently motor control. Additionally, antibiotics may stimulate the body's immune system, potentially triggering neuroinflammation. Neuroinflammation disrupts the microenvironment of nerve cells [40], affecting their survival and function, and interfering with standard motor control mechanisms, thereby increasing the risk of DIMDs. A large number of clinical case reports have shown that a certain percentage of patients treated with levofloxacin, imipenem, or ciprofloxacin for infectious diseases have developed symptoms of movement disorders consistent with the presentation of DIMDs [41–42]. These cases encompass a wide range of age groups and underlying diseases, and have been identified in healthcare facilities across various regions, indicating that this association is common and that attention should be given to the occurrence and prevention of this type of adverse reaction when antibiotics are used in clinical settings.

These findings serve as a wake-up call for clinicians when selecting relevant drugs during drug therapy, especially for patients of specific ages and genders, and also provide key clues for in-depth investigation of the pathogenesis of DIMDs and the development of targeted preventive measures. Despite the potential of the FAERS database to study DIMDs, there are limitations. First, case reports in the database may contain incomplete information and inaccurate records, which can lead to biased results in disproportionality analyses, thereby affecting accurate judgments of DIMDs associations. Second, this study focused on the association of single drugs with DIMDs and did not adequately consider drug combination use. The combined use of drugs may alter the metabolic process and pharmacodynamic properties of drugs, thereby affecting the occurrence of DIMDs; however, the study may have failed to analyse this essential factor in depth. Third, the lack of long-term follow-up data prevents a comprehensive understanding of the natural course and regression of DIMDs, which may have implications for the development of effective prevention and treatment strategies.

### Limitations

Despite the potential of the FAERS database to study DIMDs, there are limitations. First, case reports in the database may contain incomplete information and inaccurate records, which can lead to biased results of asymmetry analyses and, consequently, affect the accurate judgment of drug-DIMDs associations. Second, this study focused on the association of single drugs with DIMDs and did not adequately consider the combination of drugs. The combined use of drugs may alter the metabolic process and pharmacodynamic properties of drugs, thereby affecting the occurrence of DIMDs; however, the study may have failed to analyse this critical factor in depth. Third, the lack of long-term follow-up data prevents a comprehensive understanding of the natural course and regression of DIMDs, which may have implications for the development of effective prevention and treatment strategies. Fourth, this study focused on the association between individual drugs and DIMDs; systematic evaluation of potential drug–drug interactions was beyond the scope of the present analysis and warrants dedicated investigation in future work.

## Conclusions

This study successfully constructed a comprehensive list of drugs that may be associated with DIMDs with the help of FAERS data. The results of the study showed that age < 33 years old, males, and 62 medications, including METOCLOPRAMIDE, had a significantly higher risk of developing DIMDs. In terms of clinical practice, it can provide valuable reference information for healthcare professionals, helping to identify DIMDs at an early stage, which in turn can lead to earlier interventions and treatments, ultimately improving patient prognosis. In the field of research, the results of this study can provide key clues for future in-depth studies on the pathogenesis of DIMDs and promote research progress in this field.

## Supporting information

S1 Table Two-by-two contingency table for disproportionality analyses. (DOCX)

S2 Table Formulas and thresholds of ROR, PRR, BCPNN and EBGM. (DOCX)

S3 Table DIMDs reported in FAERS. (DOCX)

S4 Table Information related to multivariate regression. (DOCX)
