# Supplementary material for: Drug-induced movement disorder: A disproportionality analysis using the FDA adverse event reporting system (FAERS) from 2004 to 2024
Source: PLoS One. 2025 Oct 31;20(10):e0335449. doi: 10.1371/journal.pone.0335449 (PMC12578178; doi:10.1371/journal.pone.0335449)
Supplement: S1 Table — (DOCX) [file pone.0335449.s001.docx]

**S1 Table. Two-by-two contingency table for disproportionality analyses.**

|  | Target adverse events | All other adverse events | Total |
| --- | --- | --- | --- |
| Target drug | a | b | a+b |
| All other drugs | c | d | c+d |
| Total | a+c | b+d | N=a+b+c+d |
