# Supplementary material for: Drug-induced movement disorder: A disproportionality analysis using the FDA adverse event reporting system (FAERS) from 2004 to 2024
Source: PLoS One. 2025 Oct 31;20(10):e0335449. doi: 10.1371/journal.pone.0335449 (PMC12578178; doi:10.1371/journal.pone.0335449)
Supplement: S2 Table — (DOCX) [file pone.0335449.s002.docx]

**S2 Table**. **Formulas and thresholds of ROR, PRR, BCPNN and EBGM.**

| **Methods** | **Formulas** | **Thresholds** |
| --- | --- | --- |
| ROR | $\mathrm{ROR}=\frac{a/c}{b/d}$ | a ≥ 3 |
|  | $\mathrm{SE} (\mathrm{lnROR})=\sqrt{\frac{1}{a}+\frac{1}{b}+\frac{1}{c}+\frac{1}{d}}$ | 95% CI (lower limit) > 1 |
|  | $95\%\mathrm{CI}=e^{\ln(\mathrm{ROR})\pm1.96\mathrm{se}}$ |  |
| PRR | $\mathrm{PRR}=\frac{a/(a+b)}{c/(c+d)}$ | a ≥ 3 |
|  | $\mathrm{SE} (\mathrm{lnPRR})=\sqrt{\frac{1}{a}-\frac{1}{a+b}+\frac{1}{c}-\frac{1}{c+d}}$ | PRR ≥ 2 |
|  | χ2=[(ad-bc)^2](a+b+c+d)/[(a+b)(c+d)(a+c) (b+d)] | χ2≥4 |
| EBGM | $\mathrm{EBGM}=\frac{a(a+b+c+d)}{(a+c)(a+b)}$ | EBGM05 > 2 |
|  | $\mathrm{SE} (\mathrm{lnEBGM})=\sqrt{\frac{1}{a}+\frac{1}{b}+\frac{1}{c}+\frac{1}{d}}$ |  |
|  | $95\%\mathrm{CI}=e^{\ln(\mathrm{EBGM})\pm1.96\mathrm{se}}$ |  |
| BCPNN | $\mathrm{IC}=\mathrm{lo}g_{2}\frac{p (x,y)}{p (x)p (y)}=\mathrm{lo}g_{2}\frac{a(a+b+c+d)}{(a+b)(a+c)}$ | IC025 > 0 |
|  | $E (\mathrm{IC})=\mathrm{lo}g_{2}\frac{(a+\gamma11)(a+b+c+d+\alpha)(a+b+c+d+\beta)}{(a+b+c+d+\gamma)(a+b+\alpha1)(a+c+\beta1)}$ |  |
|  | $V (\mathrm{IC})=\frac{1}{{(\ln2)}^{2}}[\frac{(a+b+c+d)-a+\gamma-\gamma11}{(a+\gamma11)(1+a+b+c+d+\gamma)}+\frac{(a+b+c+d)-(a+b)+a-\alpha1}{(a+b+\alpha1)(1+a+b+c+d+\alpha)}+\frac{(a+b+c+d+\alpha)-(a+c)+\beta-\beta1}{(a+b+\beta1)(1+a+b+c+d+\beta)}]$ |  |
|  | $\gamma=\gamma11\frac{(a+b+c+d+\alpha)(a+b+c+d+\beta)}{(a+b+\alpha1)(a+c+\beta1)}$ |  |
|  | $\mathrm{IC}-2\mathrm{SD}=E (\mathrm{IC})-2\sqrt{V (IC)}$ |  |
