# Supplementary material for: Drug-induced movement disorder: A disproportionality analysis using the FDA adverse event reporting system (FAERS) from 2004 to 2024
Source: PLoS One. 2025 Oct 31;20(10):e0335449. doi: 10.1371/journal.pone.0335449 (PMC12578178; doi:10.1371/journal.pone.0335449)
Supplement: S3 Table — (DOCX) [file pone.0335449.s003.docx]

**S3 Table. DIMDs reported in FAERS**

| **Drug Category** | **DRUG** | **Number of case** | **ROR (95%Cl)** | **PRR (X²)** | **EBGM (EBGM05)** | **IC (IC025)** | **pvalue** | **p_adjust** |
| --- | --- | --- | --- | --- | --- | --- | --- | --- |
| Antipsychotic drugs | ARIPIPRAZOLE | 5860 | 12.89 ( 12.54 - 13.24 ) | 11.88 ( 56336.3 ) | 11.42 ( 11.16 ) | 3.51 ( 3.47 ) | 0 | 0 |
|  | RISPERIDONE | 4663 | 9.19 ( 8.92 - 9.47 ) | 8.68 ( 30848.63 ) | 8.42 ( 8.21 ) | 3.07 ( 3.03 ) | 0 | 0 |
|  | QUETIAPINE | 3342 | 6.15 ( 5.94 - 6.37 ) | 5.93 ( 13458.34 ) | 5.81 ( 5.64 ) | 2.54 ( 2.49 ) | 0 | 0 |
|  | VALBENAZINE | 2942 | 20.41 ( 19.62 - 21.22 ) | 17.88 ( 46239.64 ) | 17.52 ( 16.96 ) | 4.13 ( 4.07 ) | 0 | 0 |
|  | OLANZAPINE | 2251 | 6.71 ( 6.43 - 7 ) | 6.44 ( 10248.4 ) | 6.35 ( 6.13 ) | 2.67 ( 2.6 ) | 0 | 0 |
|  | PALIPERIDONE | 1740 | 6.9 ( 6.57 - 7.24 ) | 6.61 ( 8248.04 ) | 6.54 ( 6.28 ) | 2.71 ( 2.64 ) | 0 | 0 |
|  | HALOPERIDOL | 1727 | 23.04 ( 21.89 - 24.25 ) | 19.84 ( 30730.63 ) | 19.6 ( 18.78 ) | 4.29 ( 4.22 ) | 0 | 0 |
|  | ZIPRASIDONE | 1175 | 18.68 ( 17.57 - 19.86 ) | 16.53 ( 17122.07 ) | 16.4 ( 15.58 ) | 4.04 ( 3.95 ) | 0 | 0 |
|  | LURASIDONE | 1076 | 10.65 ( 10.01 - 11.34 ) | 9.94 ( 8653.15 ) | 9.87 ( 9.37 ) | 3.3 ( 3.21 ) | 0 | 0 |
|  | BREXPIPRAZOLE | 902 | 12.35 ( 11.54 - 13.23 ) | 11.4 ( 8563.74 ) | 11.33 ( 10.7 ) | 3.5 ( 3.4 ) | 0 | 0 |
|  | TETRABENAZINE | 592 | 12.1 ( 11.12 - 13.16 ) | 11.18 ( 5504.93 ) | 11.14 ( 10.38 ) | 3.48 ( 3.35 ) | 0 | 0 |
|  | DEUTETRABENAZINE | 556 | 18.21 ( 16.67 - 19.9 ) | 16.16 ( 7933.76 ) | 16.1 ( 14.95 ) | 4.01 ( 3.88 ) | 0 | 0 |
|  | CARIPRAZINE | 523 | 15.21 ( 13.89 - 16.65 ) | 13.76 ( 6213.67 ) | 13.72 ( 12.72 ) | 3.78 ( 3.65 ) | 0 | 0 |
|  | ASENAPINE | 413 | 8.7 ( 7.87 - 9.61 ) | 8.23 ( 2634.69 ) | 8.21 ( 7.55 ) | 3.04 ( 2.89 ) | 0 | 0 |
|  | LUMATEPERONE | 209 | 8.26 ( 7.18 - 9.5 ) | 7.84 ( 1254.37 ) | 7.83 ( 6.96 ) | 2.97 ( 2.76 ) | 2.9E-273 | 5.1E-270 |
|  | PROCHLORPERAZINE | 76 | 21.69 ( 17.02 - 27.65 ) | 18.81 ( 1290.34 ) | 18.8 ( 15.35 ) | 4.23 ( 3.88 ) | 8.81E-70 | 1.56E-66 |
|  | FLUPHENAZINE | 64 | 20.74 ( 15.94 - 26.99 ) | 18.09 ( 1040.86 ) | 18.09 ( 14.51 ) | 4.18 ( 3.8 ) | 5.99E-58 | 1.06E-54 |
|  | ILOPERIDONE | 54 | 7.8 ( 5.93 - 10.26 ) | 7.42 ( 302.24 ) | 7.42 ( 5.9 ) | 2.89 ( 2.49 ) | 2.7E-66 | 4.79E-63 |
|  | CHLORPROMAZINE | 51 | 10.62 ( 7.98 - 14.12 ) | 9.91 ( 411.44 ) | 9.91 ( 7.8 ) | 3.31 ( 2.89 ) | 1.56E-89 | 2.76E-86 |
|  | PERPHENAZINE | 42 | 28.54 ( 20.45 - 39.82 ) | 23.7 ( 919.57 ) | 23.69 ( 17.93 ) | 4.57 ( 4.09 ) | 1.02E-43 | 1.81E-40 |
|  | TIOTIXENE | 37 | 25.27 ( 17.78 - 35.9 ) | 21.41 ( 725.17 ) | 21.41 ( 15.95 ) | 4.42 ( 3.92 ) | 5.29E-37 | 9.38E-34 |
|  | PIMOZIDE | 32 | 30.37 ( 20.69 - 44.59 ) | 24.94 ( 740.71 ) | 24.94 ( 18.09 ) | 4.64 ( 4.09 ) | 1.88E-34 | 3.34E-31 |
|  | OLANZAPINE;SAMIDORPHAN | 27 | 3.58 ( 2.45 - 5.25 ) | 3.52 ( 48.99 ) | 3.52 ( 2.55 ) | 1.81 ( 1.26 ) | 9.21E-12 | 1.63E-08 |
|  | THIORIDAZINE | 17 | 11.73 ( 7.14 - 19.25 ) | 10.86 ( 153.34 ) | 10.86 ( 7.17 ) | 3.44 ( 2.73 ) | 7.55E-13 | 1.34E-09 |
|  | DROPERIDOL | 15 | 11.67 ( 6.88 - 19.78 ) | 10.81 ( 134.59 ) | 10.81 ( 6.95 ) | 3.43 ( 2.68 ) | 1.76E-11 | 3.11E-08 |
|  | THIETHYLPERAZINE | 13 | 64.43 ( 33.25 - 124.87 ) | 43.82 ( 547.91 ) | 43.81 ( 25.19 ) | 5.45 ( 4.56 ) | 2.06E-18 | 3.64E-15 |
|  | LOXAPINE | 11 | 18.63 ( 9.92 - 35.01 ) | 16.48 ( 161.1 ) | 16.48 ( 9.72 ) | 4.04 ( 3.16 ) | 9.09E-11 | 1.61E-07 |
|  | TRIFLUOPERAZINE | 9 | 10.66 ( 5.41 - 21.01 ) | 9.95 ( 72.95 ) | 9.94 ( 5.63 ) | 3.31 ( 2.37 ) | 3.9E-07 | 0.000691 |
|  | AMISULPRIDE | 6 | 7.23 ( 3.18 - 16.45 ) | 6.91 ( 30.57 ) | 6.91 ( 3.48 ) | 2.79 ( 1.67 ) | 0.000258 | 0.457117 |
|  | MOLINDONE | 5 | 74.34 ( 24.91 - 221.82 ) | 48.15 ( 232.55 ) | 48.14 ( 19.29 ) | 5.59 ( 4.2 ) | 4.25E-08 | 7.53E-05 |
|  | PIPAMPERONE | 4 | 9.56 ( 3.47 - 26.36 ) | 8.99 ( 28.61 ) | 8.99 ( 3.85 ) | 3.17 ( 1.83 ) | 0.001061 | 1 |
|  | LEVOMEPROMAZINE | 3 | 57.35 ( 14.83 - 221.77 ) | 40.44 ( 116.26 ) | 40.44 ( 13.04 ) | 5.34 ( 3.65 ) | 4.71E-05 | 0.083432 |
| Anti-parkinson's disease drugs | CARBIDOPA;LEVODOPA | 5414 | 15.92 ( 15.47 - 16.38 ) | 14.39 ( 65271.73 ) | 13.86 ( 13.53 ) | 3.79 ( 3.75 ) | 0 | 0 |
|  | PRAMIPEXOLE | 502 | 8.12 ( 7.42 - 8.89 ) | 7.72 ( 2946.63 ) | 7.69 ( 7.13 ) | 2.94 ( 2.81 ) | 0 | 0 |
|  | ROPINIROLE | 473 | 8.65 ( 7.88 - 9.5 ) | 8.19 ( 2997.59 ) | 8.17 ( 7.55 ) | 3.03 ( 2.89 ) | 0 | 0 |
|  | ISTRADEFYLLINE | 443 | 14.51 ( 13.15 - 16.01 ) | 13.19 ( 5013.24 ) | 13.15 ( 12.12 ) | 3.72 ( 3.57 ) | 0 | 0 |
|  | APOMORPHINE | 366 | 6.51 ( 5.86 - 7.23 ) | 6.26 ( 1623.74 ) | 6.24 ( 5.72 ) | 2.64 ( 2.49 ) | 0 | 0 |
|  | LEVODOPA | 314 | 8.36 ( 7.46 - 9.37 ) | 7.93 ( 1911.67 ) | 7.91 ( 7.19 ) | 2.98 ( 2.82 ) | 0 | 0 |
|  | ROTIGOTINE | 277 | 4.72 ( 4.19 - 5.32 ) | 4.59 ( 782.63 ) | 4.59 ( 4.15 ) | 2.2 ( 2.02 ) | 2E-171 | 3.5E-168 |
|  | ENTACAPONE | 249 | 22.64 ( 19.79 - 25.9 ) | 19.51 ( 4398.68 ) | 19.48 ( 17.41 ) | 4.28 ( 4.09 ) | 0 | 0 |
|  | AMANTADINE | 226 | 11.13 ( 9.71 - 12.74 ) | 10.35 ( 1919.99 ) | 10.33 ( 9.22 ) | 3.37 ( 3.17 ) | 0 | 0 |
|  | CARBIDOPA;ENTACAPONE;LEVODOPA | 187 | 11.48 ( 9.88 - 13.33 ) | 10.65 ( 1645.02 ) | 10.64 ( 9.39 ) | 3.41 ( 3.19 ) | 0 | 0 |
|  | RASAGILINE | 171 | 9 ( 7.71 - 10.5 ) | 8.49 ( 1137.56 ) | 8.48 ( 7.45 ) | 3.08 ( 2.86 ) | 9.8E-248 | 1.7E-244 |
|  | OPICAPONE | 119 | 22.41 ( 18.46 - 27.22 ) | 19.34 ( 2083.67 ) | 19.33 ( 16.43 ) | 4.27 ( 3.99 ) | 0 | 0 |
|  | CARBIDOPA | 80 | 8.21 ( 6.55 - 10.29 ) | 7.79 ( 476.77 ) | 7.79 ( 6.45 ) | 2.96 ( 2.63 ) | 3.3E-104 | 5.8E-101 |
|  | BENZATROPINE | 44 | 14.09 ( 10.33 - 19.22 ) | 12.84 ( 483.93 ) | 12.84 ( 9.9 ) | 3.68 ( 3.23 ) | 7.43E-34 | 1.32E-30 |
|  | FOSCARBIDOPA;FOSLEVODOPA | 36 | 17.85 ( 12.6 - 25.27 ) | 15.86 ( 504.96 ) | 15.86 ( 11.85 ) | 3.99 ( 3.49 ) | 2.91E-31 | 5.16E-28 |
|  | SAFINAMIDE | 30 | 16.66 ( 11.4 - 24.35 ) | 14.93 ( 392.62 ) | 14.92 ( 10.86 ) | 3.9 ( 3.35 ) | 1.57E-25 | 2.78E-22 |
|  | BROMOCRIPTINE | 28 | 3.55 ( 2.44 - 5.16 ) | 3.48 ( 49.83 ) | 3.48 ( 2.54 ) | 1.8 ( 1.26 ) | 5.9E-12 | 1.04E-08 |
|  | TRIHEXYPHENIDYL | 16 | 14.18 ( 8.47 - 23.74 ) | 12.92 ( 177.2 ) | 12.92 ( 8.39 ) | 3.69 ( 2.96 ) | 2.43E-13 | 4.31E-10 |
|  | PERGOLIDE | 15 | 6.65 ( 3.96 - 11.16 ) | 6.38 ( 68.54 ) | 6.38 ( 4.13 ) | 2.67 ( 1.93 ) | 2.49E-08 | 4.41E-05 |
|  | BIPERIDEN | 8 | 4.3 ( 2.13 - 8.69 ) | 4.2 ( 19.62 ) | 4.2 ( 2.33 ) | 2.07 ( 1.09 ) | 0.000761 | 1 |
|  | PROCYCLIDINE | 7 | 31.22 ( 13.71 - 71.08 ) | 25.5 ( 166.03 ) | 25.5 ( 12.81 ) | 4.67 ( 3.55 ) | 1.05E-08 | 1.85E-05 |
|  | TOLCAPONE | 4 | 7.76 ( 2.83 - 21.26 ) | 7.39 ( 22.25 ) | 7.39 ( 3.18 ) | 2.88 ( 1.55 ) | 0.002194 | 1 |
| Antidepressant drugs | SERTRALINE | 1467 | 3.93 ( 3.73 - 4.14 ) | 3.85 ( 3080.84 ) | 3.82 ( 3.65 ) | 1.93 ( 1.86 ) | 0 | 0 |
|  | DULOXETINE | 1123 | 2.89 ( 2.73 - 3.07 ) | 2.85 ( 1349.71 ) | 2.84 ( 2.7 ) | 1.5 ( 1.42 ) | 4.5E-295 | 8E-292 |
|  | PAROXETINE | 1019 | 3.6 ( 3.38 - 3.83 ) | 3.53 ( 1851.7 ) | 3.52 ( 3.34 ) | 1.81 ( 1.72 ) | 0 | 0 |
|  | VENLAFAXINE | 855 | 2.8 ( 2.61 - 2.99 ) | 2.76 ( 961.44 ) | 2.75 ( 2.6 ) | 1.46 ( 1.36 ) | 1E-210 | 1.9E-207 |
|  | FLUOXETINE | 800 | 4.4 ( 4.1 - 4.72 ) | 4.29 ( 2020.19 ) | 4.27 ( 4.02 ) | 2.09 ( 1.99 ) | 0 | 0 |
|  | CITALOPRAM | 678 | 3.74 ( 3.46 - 4.04 ) | 3.67 ( 1317.77 ) | 3.65 ( 3.43 ) | 1.87 ( 1.76 ) | 5.9E-288 | 1.1E-284 |
|  | ESCITALOPRAM | 564 | 3.5 ( 3.22 - 3.8 ) | 3.43 ( 975.85 ) | 3.42 ( 3.19 ) | 1.78 ( 1.65 ) | 1.1E-213 | 1.9E-210 |
|  | ATOMOXETINE | 540 | 4.16 ( 3.82 - 4.54 ) | 4.07 ( 1253.58 ) | 4.06 ( 3.77 ) | 2.02 ( 1.89 ) | 6.5E-274 | 1.1E-270 |
|  | MIRTAZAPINE | 484 | 3.59 ( 3.28 - 3.93 ) | 3.52 ( 877.5 ) | 3.51 ( 3.26 ) | 1.81 ( 1.68 ) | 2.7E-192 | 4.8E-189 |
|  | VILAZODONE | 116 | 2.89 ( 2.4 - 3.47 ) | 2.85 ( 139.95 ) | 2.85 ( 2.44 ) | 1.51 ( 1.24 ) | 6.95E-32 | 1.23E-28 |
|  | TRAZODONE | 98 | 2.45 ( 2.01 - 3 ) | 2.43 ( 82.8 ) | 2.43 ( 2.05 ) | 1.28 ( 0.99 ) | 1.87E-19 | 3.31E-16 |
|  | AMITRIPTYLINE | 96 | 2.69 ( 2.2 - 3.3 ) | 2.66 ( 100.18 ) | 2.66 ( 2.25 ) | 1.41 ( 1.12 ) | 3.23E-23 | 5.71E-20 |
|  | FLUVOXAMINE | 50 | 5.22 ( 3.94 - 6.92 ) | 5.06 ( 164.12 ) | 5.06 ( 3.99 ) | 2.34 ( 1.93 ) | 1.1E-36 | 1.95E-33 |
|  | CLOMIPRAMINE | 44 | 4.89 ( 3.62 - 6.61 ) | 4.75 ( 131.36 ) | 4.75 ( 3.69 ) | 2.25 ( 1.81 ) | 1.37E-29 | 2.42E-26 |
|  | ZURANOLONE | 23 | 8.67 ( 5.69 - 13.22 ) | 8.2 ( 146.55 ) | 8.2 ( 5.76 ) | 3.04 ( 2.43 ) | 3.1E-14 | 5.5E-11 |
|  | IMIPRAMINE | 21 | 3.31 ( 2.15 - 5.1 ) | 3.25 ( 33.03 ) | 3.25 ( 2.26 ) | 1.7 ( 1.08 ) | 2.86E-08 | 5.06E-05 |
|  | MAPROTILINE | 7 | 5.85 ( 2.75 - 12.48 ) | 5.65 ( 26.99 ) | 5.65 ( 3 ) | 2.5 ( 1.45 ) | 0.000278 | 0.493125 |
|  | TIANEPTINE | 4 | 8.77 ( 3.19 - 24.13 ) | 8.3 ( 25.86 ) | 8.3 ( 3.56 ) | 3.05 ( 1.71 ) | 0.00143 | 1 |
| Antiepileptic drugs | PREGABALIN | 2010 | 2.39 ( 2.29 - 2.5 ) | 2.37 ( 1573.63 ) | 2.35 ( 2.26 ) | 1.23 ( 1.16 ) | 0 | 0 |
|  | GABAPENTIN | 1270 | 2.7 ( 2.55 - 2.85 ) | 2.67 ( 1319.42 ) | 2.65 ( 2.53 ) | 1.41 ( 1.32 ) | 1.6E-288 | 2.8E-285 |
|  | LAMOTRIGINE | 1037 | 2.73 ( 2.57 - 2.91 ) | 2.7 ( 1107.08 ) | 2.68 ( 2.55 ) | 1.42 ( 1.33 ) | 2.2E-242 | 4E-239 |
|  | VALPROIC ACID | 971 | 3.91 ( 3.67 - 4.17 ) | 3.83 ( 2031.74 ) | 3.81 ( 3.61 ) | 1.93 ( 1.84 ) | 0 | 0 |
|  | CLONAZEPAM | 469 | 3.34 ( 3.05 - 3.66 ) | 3.29 ( 749.02 ) | 3.28 ( 3.04 ) | 1.71 ( 1.58 ) | 2.1E-164 | 3.7E-161 |
|  | CARBAMAZEPINE | 401 | 2.57 ( 2.33 - 2.84 ) | 2.54 ( 377.16 ) | 2.54 ( 2.34 ) | 1.34 ( 1.2 ) | 1.12E-83 | 1.99E-80 |
|  | OXCARBAZEPINE | 320 | 4.83 ( 4.32 - 5.4 ) | 4.69 ( 934.99 ) | 4.69 ( 4.27 ) | 2.23 ( 2.06 ) | 1.6E-204 | 2.8E-201 |
|  | LORAZEPAM | 285 | 2.6 ( 2.31 - 2.92 ) | 2.57 ( 274.36 ) | 2.56 ( 2.33 ) | 1.36 ( 1.19 ) | 2.8E-61 | 4.96E-58 |
|  | PHENYTOIN | 281 | 2.47 ( 2.2 - 2.78 ) | 2.45 ( 241.82 ) | 2.44 ( 2.21 ) | 1.29 ( 1.12 ) | 3.28E-54 | 5.81E-51 |
|  | VIGABATRIN | 226 | 2.38 ( 2.09 - 2.72 ) | 2.36 ( 178.24 ) | 2.36 ( 2.11 ) | 1.24 ( 1.04 ) | 2.34E-40 | 4.14E-37 |
|  | CLOBAZAM | 124 | 3.23 ( 2.7 - 3.86 ) | 3.18 ( 186.38 ) | 3.18 ( 2.74 ) | 1.67 ( 1.41 ) | 5.89E-42 | 1.04E-38 |
|  | TIAGABINE | 22 | 8.87 ( 5.76 - 13.65 ) | 8.38 ( 144.01 ) | 8.38 ( 5.84 ) | 3.07 ( 2.45 ) | 7.24E-14 | 1.28E-10 |
|  | RUFINAMIDE | 10 | 3.76 ( 2 - 7.05 ) | 3.68 ( 19.69 ) | 3.68 ( 2.18 ) | 1.88 ( 1 ) | 0.000489 | 0.866823 |
| Gastrointestinal motility drugs | TROFINETIDE | 156 | 7.83 ( 6.67 - 9.21 ) | 7.46 ( 877.69 ) | 7.45 ( 6.51 ) | 2.9 ( 2.66 ) | 1.8E-191 | 3.2E-188 |
|  | METOCLOPRAMIDE | 18266 | 766.5(739.48-794.51 ) | 129.19 ( 2031083.38 ) | 112.23 ( 108.91 ) | 6.81 ( 6.78 ) | 0 | 0 |
|  | ONDANSETRON | 399 | 4.33 ( 3.92 - 4.79 ) | 4.23 ( 988.3 ) | 4.22 ( 3.88 ) | 2.08 ( 1.93 ) | 3.2E-216 | 5.6E-213 |
|  | PALONOSETRON | 16 | 4.67 ( 2.84 - 7.7 ) | 4.55 ( 44.65 ) | 4.55 ( 3 ) | 2.19 ( 1.47 ) | 8.32E-07 | 0.001474 |
|  | DOMPERIDONE | 7 | 8.01 ( 3.73 - 17.16 ) | 7.61 ( 40.49 ) | 7.61 ( 4.02 ) | 2.93 ( 1.88 ) | 4.36E-05 | 0.077281 |
|  | DOLASETRON | 4 | 10.49 ( 3.79 - 29.04 ) | 9.8 ( 31.86 ) | 9.8 ( 4.18 ) | 3.29 ( 1.95 ) | 0.000764 | 1 |
| The central nervous system stimulants | METHYLPHENIDATE | 1122 | 4 ( 3.77 - 4.24 ) | 3.91 ( 2430.83 ) | 3.89 ( 3.7 ) | 1.96 ( 1.87 ) | 0 | 0 |
|  | LISDEXAMFETAMINE | 558 | 4.33 ( 3.98 - 4.71 ) | 4.22 ( 1378.16 ) | 4.21 ( 3.92 ) | 2.07 ( 1.95 ) | 5.9E-301 | 1E-297 |
|  | DEXMETHYLPHENIDATE | 84 | 5.67 ( 4.56 - 7.05 ) | 5.48 ( 309.62 ) | 5.48 ( 4.56 ) | 2.45 ( 2.13 ) | 2.51E-68 | 4.45E-65 |
|  | MODAFINIL | 73 | 2.95 ( 2.34 - 3.72 ) | 2.91 ( 92.14 ) | 2.91 ( 2.4 ) | 1.54 ( 1.2 ) | 2.12E-21 | 3.75E-18 |
|  | DEXMETHYLPHENIDATE;SERDEXMETHYLPHENIDATE | 21 | 7.15 ( 4.61 - 11.09 ) | 6.84 ( 105.45 ) | 6.84 ( 4.74 ) | 2.77 ( 2.14 ) | 1.23E-11 | 2.19E-08 |
| Antibiotics | CIPROFLOXACIN | 1068 | 3.9 ( 3.67 - 4.15 ) | 3.82 ( 2225.08 ) | 3.8 ( 3.61 ) | 1.93 ( 1.84 ) | 0 | 0 |
|  | LEVOFLOXACIN | 733 | 2.71 ( 2.52 - 2.92 ) | 2.68 ( 772.54 ) | 2.67 ( 2.51 ) | 1.42 ( 1.31 ) | 1.2E-169 | 2.1E-166 |
|  | CILASTATIN;IMIPENEM | 135 | 4.5 ( 3.79 - 5.34 ) | 4.38 ( 354.83 ) | 4.38 ( 3.79 ) | 2.13 ( 1.88 ) | 2.07E-78 | 3.67E-75 |
|  | CEFEPIME | 66 | 4.74 ( 3.71 - 6.06 ) | 4.61 ( 187.83 ) | 4.61 ( 3.75 ) | 2.2 ( 1.85 ) | 5.82E-42 | 1.03E-38 |
| Anti-dementia drugs | RIVASTIGMINE | 269 | 2.53 ( 2.24 - 2.85 ) | 2.5 ( 243 ) | 2.5 ( 2.26 ) | 1.32 ( 1.14 ) | 1.85E-54 | 3.28E-51 |
|  | DONEPEZIL | 259 | 4.07 ( 3.6 - 4.61 ) | 3.98 ( 581.21 ) | 3.97 ( 3.58 ) | 1.99 ( 1.81 ) | 9.2E-128 | 1.6E-124 |
|  | MEMANTINE | 218 | 3.28 ( 2.87 - 3.76 ) | 3.23 ( 337.55 ) | 3.23 ( 2.88 ) | 1.69 ( 1.49 ) | 6.7E-75 | 1.19E-71 |
|  | XANOMELINE | 7 | 5.97 ( 2.8 - 12.72 ) | 5.75 ( 27.7 ) | 5.75 ( 3.05 ) | 2.52 ( 1.48 ) | 0.000249 | 0.441727 |
| Local anaesthetic drugs | ROPIVACAINE | 54 | 4.51 ( 3.44 - 5.91 ) | 4.39 ( 142.59 ) | 4.39 ( 3.5 ) | 2.14 ( 1.74 ) | 3.99E-32 | 7.07E-29 |
|  | MEPIVACAINE | 18 | 3.23 ( 2.03 - 5.16 ) | 3.18 ( 27.11 ) | 3.18 ( 2.15 ) | 1.67 ( 1 ) | 5.87E-07 | 0.00104 |
|  | LEVOBUPIVACAINE | 8 | 9.15 ( 4.47 - 18.73 ) | 8.63 ( 54.35 ) | 8.63 ( 4.74 ) | 3.11 ( 2.11 ) | 4.99E-06 | 0.008841 |
|  | BUPIVACAINE;EPINEPHRINE | 7 | 4.24 ( 2 - 8.99 ) | 4.14 ( 16.79 ) | 4.14 ( 2.21 ) | 2.05 ( 1.01 ) | 0.001728 | 1 |
| Analgesic drugs | DEXTROPROPOXYPHENE;PARACETAMOL | 1301 | 79.22 ( 73.97 - 84.85 ) | 50.3 ( 62740.99 ) | 49.84 ( 47.06 ) | 5.64 ( 5.55 ) | 0 | 0 |
|  | NALOXONE;OXYCODONE | 13 | 4.38 ( 2.52 - 7.61 ) | 4.27 ( 32.85 ) | 4.27 ( 2.69 ) | 2.1 ( 1.31 ) | 1.66E-05 | 0.029376 |
|  | NALOXONE;PENTAZOCINE | 3 | 11.47 ( 3.53 - 37.29 ) | 10.64 ( 26.41 ) | 10.64 ( 3.97 ) | 3.41 ( 1.9 ) | 0.002836 | 1 |
| Contrast agents | GADOBENIC ACID | 99 | 3.96 ( 3.25 - 4.84 ) | 3.88 ( 212.9 ) | 3.88 ( 3.28 ) | 1.95 ( 1.66 ) | 1.37E-47 | 2.42E-44 |
|  | GADOTERIC ACID | 58 | 2.69 ( 2.07 - 3.48 ) | 2.65 ( 60.15 ) | 2.65 ( 2.13 ) | 1.41 ( 1.03 ) | 2.03E-14 | 3.6E-11 |
|  | GADOLINIUM | 29 | 4 ( 2.76 - 5.78 ) | 3.91 ( 63.26 ) | 3.91 ( 2.87 ) | 1.97 ( 1.43 ) | 7.88E-15 | 1.4E-11 |
| Nutritional supplements | DOCOSAHEXAENOIC  ACID;EICOSAPENTAENOIC  ACID;PHOSPHATIDYL SERINE | 20 | 6.46 ( 4.13 - 10.13 ) | 6.21 ( 88.12 ) | 6.21 ( 4.27 ) | 2.64 ( 1.99 ) | 2.02E-10 | 3.58E-07 |
|  | CYANOCOBALAMIN | 19 | 4.03 ( 2.55 - 6.36 ) | 3.94 ( 42 ) | 3.94 ( 2.69 ) | 1.98 ( 1.32 ) | 7.16E-07 | 0.001267 |
| Cannabinoid drugs | 8-TETRAHYDROCANNABINOL | 22 | 10.86 ( 7.04 - 16.78 ) | 10.12 ( 182.21 ) | 10.12 ( 7.04 ) | 3.34 ( 2.71 ) | 1.51E-15 | 2.67E-12 |
|  | NABILONE | 6 | 10.04 ( 4.38 - 23.01 ) | 9.41 ( 45.4 ) | 9.4 ( 4.7 ) | 3.23 ( 2.1 ) | 4.72E-05 | 0.083517 |
| Anti-anxiety drugs | BUSPIRONE | 87 | 6.47 ( 5.22 - 8.03 ) | 6.22 ( 383.88 ) | 6.22 ( 5.19 ) | 2.64 ( 2.32 ) | 2.46E-84 | 4.35E-81 |
|  | MEPROBAMATE | 4 | 5.52 ( 2.03 - 15 ) | 5.34 ( 14.21 ) | 5.34 ( 2.31 ) | 2.42 ( 1.09 ) | 0.006991 | 1 |
| antihistamine drugs | PROMETHAZINE | 135 | 5.33 ( 4.49 - 6.33 ) | 5.17 ( 456.59 ) | 5.16 ( 4.47 ) | 2.37 ( 2.12 ) | 2.2E-100 | 3.82E-97 |
|  | DIMENHYDRINATE | 8 | 4.56 ( 2.25 - 9.22 ) | 4.44 ( 21.47 ) | 4.44 ( 2.46 ) | 2.15 ( 1.17 ) | 0.000528 | 0.935929 |
| Ophthalmic drugs | DICLOFENAMIDE | 32 | 2.87 ( 2.02 - 4.07 ) | 2.83 ( 38.06 ) | 2.83 ( 2.11 ) | 1.5 ( 0.99 ) | 1.74E-09 | 3.08E-06 |
|  | CYCLOPENTOLATE | 18 | 5.06 ( 3.16 - 8.1 ) | 4.91 ( 56.5 ) | 4.91 ( 3.31 ) | 2.3 ( 1.62 ) | 5.74E-08 | 0.000102 |
| Antacid drugs | SODIUM CITRATE | 124 | 2.52 ( 2.11 - 3.01 ) | 2.49 ( 111.24 ) | 2.49 ( 2.14 ) | 1.32 ( 1.05 ) | 1.12E-25 | 1.98E-22 |
| Laxative drugs | MACROGOL | 11 | 4.1 ( 2.25 - 7.47 ) | 4.01 ( 25.02 ) | 4.01 ( 2.43 ) | 2 ( 1.16 ) | 0.000127 | 0.224724 |
| Electrolyte balance regulating drugs | ASPARTIC ACID;CAFFEINE;HONEY;INOSITOL;MAGNESIUM;MALIC ACID;PYRIDOXINE;RIBOFLAVIN;SUCROSE;TAURINE;THIAMINE | 3 | 11.47 ( 3.53 - 37.29 ) | 10.64 ( 26.41 ) | 10.64 ( 3.97 ) | 3.41 ( 1.9 ) | 0.002836 | 1 |
| Disinfectant and antiseptic drugs | PHENOL | 4 | 12.16 ( 4.37 - 33.86 ) | 11.23 ( 37.57 ) | 11.23 ( 4.77 ) | 3.49 ( 2.14 ) | 0.000454089 | 0.804192174 |
| Oral contraceptive drugs | ETHINYLESTRADIOL;GESTODENE | 4 | 5.95 ( 2.18 - 16.19 ) | 5.74 ( 15.76 ) | 5.74 ( 2.48 ) | 2.52 ( 1.2 ) | 0.00543782 | 1 |
| Immune serum and toxin neutralizing drugs | IMMUNOGLOBULIN HUMAN ANTI-RABIES | 5 | 5.87 ( 2.4 - 14.37 ) | 5.66 ( 19.35 ) | 5.66 ( 2.68 ) | 2.5 ( 1.29 ) | 0.002038323 | 1 |
| Vaccine | PNEUMOCOCCAL VACCINE | 4 | 7.33 ( 2.68 - 20.06 ) | 7 ( 20.74 ) | 7 ( 3.02 ) | 2.81 ( 1.48 ) | 0.002664352 | 1 |
| Anti-tumor drugs | NELARABINE | 17 | 4.37 ( 2.7 - 7.09 ) | 4.27 ( 42.85 ) | 4.27 ( 2.85 ) | 2.09 ( 1.4 ) | 9.21623E-07 | 0.001632194 |
| General anesthetic drugs | PROPOFOL | 158 | 2.78 ( 2.37 - 3.25 ) | 2.74 ( 175.69 ) | 2.74 ( 2.4 ) | 1.45 ( 1.22 ) | 1.01822E-39 | 1.80327E-36 |
| Antiasthmatic drugs | MONTELUKAST | 631 | 4.02 ( 3.71 - 4.35 ) | 3.93 ( 1381.94 ) | 3.92 ( 3.66 ) | 1.97 ( 1.85 ) | 7.6789E-302 | 1.3599E-298 |
| Antimalarial drugs | MEFLOQUINE | 26 | 3.14 ( 2.13 - 4.63 ) | 3.09 ( 36.99 ) | 3.09 ( 2.23 ) | 1.63 ( 1.06 ) | 3.45056E-09 | 6.11095E-06 |
| Antimanic drugs | LITHIUM | 257 | 5.08 ( 4.49 - 5.76 ) | 4.93 ( 810.31 ) | 4.93 ( 4.44 ) | 2.3 ( 2.12 ) | 2.2401E-177 | 3.9673E-174 |
| Appetite suppressant drugs | FENFLURAMINE | 85 | 4.5 ( 3.63 - 5.59 ) | 4.39 ( 223.95 ) | 4.39 ( 3.66 ) | 2.13 ( 1.82 ) | 6.86134E-50 | 1.21514E-46 |
| Antihypertensive drugs | GUANFACINE | 77 | 3.56 ( 2.84 - 4.46 ) | 3.49 ( 137.94 ) | 3.49 ( 2.89 ) | 1.8 ( 1.47 ) | 2.6466E-31 | 4.68713E-28 |
| Skeletal muscle relaxant drugs | TIZANIDINE | 44 | 2.85 ( 2.11 - 3.84 ) | 2.81 ( 51.58 ) | 2.81 ( 2.19 ) | 1.49 ( 1.05 ) | 1.72795E-12 | 3.0602E-09 |
| Anticholinesterase drugs | PYRIDOSTIGMINE | 29 | 4.09 ( 2.82 - 5.91 ) | 3.99 ( 65.55 ) | 3.99 ( 2.93 ) | 2 ( 1.46 ) | 2.56648E-15 | 4.54523E-12 |
| Other drugs | BACLOFEN | 576 | 2.74 ( 2.52 - 2.98 ) | 2.71 ( 621.56 ) | 2.7 ( 2.52 ) | 1.43 ( 1.31 ) | 8.0918E-137 | 1.4331E-133 |
|  | FLUOXETINE;OLANZAPINE | 15 | 3.35 ( 2 - 5.58 ) | 3.29 ( 24.06 ) | 3.29 ( 2.14 ) | 1.72 ( 0.99 ) | 7.85533E-05 | 0.13911793 |
|  | CERLIPONASE ALFA | 13 | 4.2 ( 2.42 - 7.3 ) | 4.1 ( 30.75 ) | 4.1 ( 2.59 ) | 2.04 ( 1.25 ) | 2.52552E-05 | 0.0447269 |
|  | AMIFAMPRIDINE | 39 | 3.02 ( 2.2 - 4.14 ) | 2.97 ( 51.36 ) | 2.97 ( 2.28 ) | 1.57 ( 1.11 ) | 2.09738E-12 | 3.71445E-09 |
|  | MIGLUSTAT | 25 | 3.49 ( 2.35 - 5.19 ) | 3.43 ( 43.33 ) | 3.43 ( 2.46 ) | 1.78 ( 1.2 ) | 1.59105E-10 | 2.81774E-07 |
|  | METHYLTHIONINIUM | 15 | 6.43 ( 3.83 - 10.8 ) | 6.18 ( 65.67 ) | 6.18 ( 4.01 ) | 2.63 ( 1.89 ) | 3.74191E-08 | 6.62692E-05 |
|  | ATROPA BELLA-DONNA;CALCIUM PHOSPHATE;COFFEA ARABICA;MATRICARIA CHAMOMILLA | 9 | 9.34 ( 4.75 - 18.35 ) | 8.79 ( 62.61 ) | 8.79 ( 4.99 ) | 3.14 ( 2.19 ) | 1.10264E-06 | 0.001952777 |
|  | CEVIMELINE | 9 | 4.61 ( 2.37 - 8.97 ) | 4.49 ( 24.63 ) | 4.49 ( 2.58 ) | 2.17 ( 1.24 ) | 0.000221112 | 0.391588575 |
