# Supplementary material for: Drug-induced movement disorder: A disproportionality analysis using the FDA adverse event reporting system (FAERS) from 2004 to 2024
Source: PLoS One. 2025 Oct 31;20(10):e0335449. doi: 10.1371/journal.pone.0335449 (PMC12578178; doi:10.1371/journal.pone.0335449)
Supplement: S4 Table — (DOCX) [file pone.0335449.s004.docx]

**S4 Table. Information related to multivariate regression**

| **Variables** | **OR(95%CI)** | **Pvalue** | **Padjust** | **Padjust＜0.01** |
| --- | --- | --- | --- | --- |
| Q2 (33~53) | Reference |  |  |  |
| Q1(＜33） | 1.3698(1.3305, 1.4104) | 2.02E-99 | 8.09E-99 | YES |
| Q3 (53~67) | 0.7294(0.7051, 0.7545) | 1.09E-74 | 4.37E-74 | YES |
| Q4 (＞67) | 0.6928(0.669, 0.7174) | 3.56E-94 | 1.42E-93 | YES |
| Female | Reference |  |  |  |
| Male | 1.036(1.0122, 1.0603) | 0.002838485 | 0.011353939 | P＜0.05 |
| METOCLOPRAMIDE | 103.5965(91.8544, 116.6949) | 0 | 0 | YES |
| ARIPIPRAZOLE | 18.0311(16.8339, 19.2931) | 0 | 0 | YES |
| CARBIDOPA;LEVODOPA | 36.9697(34.6073, 39.4608) | 0 | 0 | YES |
| RISPERIDONE | 16.2892(15.0126, 17.6463) | 0 | 0 | YES |
| QUETIAPINE | 10.2066(9.5452, 10.9015) | 0 | 0 | YES |
| VALBENAZINE | 42.6696(32.4905, 55.204) | 3.80E-170 | 3.76E-168 | YES |
| OLANZAPINE | 9.3944(8.5492, 10.2984) | 0 | 0 | YES |
| PREGABALIN | 5.0594(4.7237, 5.4122) | 0 | 0 | YES |
| PALIPERIDONE | 14.8332(13.2878, 16.5072) | 0 | 0 | YES |
| HALOPERIDOL | 67.4234(61.9025, 73.3641) | 0 | 0 | YES |
| SERTRALINE | 6.4846(5.9403, 7.0638) | 0 | 0 | YES |
| GABAPENTIN | 5.7618(5.2199, 6.3423) | 2.36E-272 | 2.33E-270 | YES |
| ZIPRASIDONE | 24.0898(21.1896, 27.282) | 0 | 0 | YES |
| DULOXETINE | 6.2201(5.5208, 6.9794) | 2.61E-205 | 2.58E-203 | YES |
| METHYLPHENIDATE | 6.7311(5.9798, 7.5491) | 6.57E-226 | 6.51E-224 | YES |
| LURASIDONE | 23.0859(19.7836, 26.7872) | 0 | 0 | YES |
| CIPROFLOXACIN | 10.354(9.5731, 11.1805) | 0 | 0 | YES |
| LAMOTRIGINE | 4.6742(4.0621, 5.3481) | 3.56E-107 | 3.53E-105 | YES |
| PAROXETINE | 8.0936(7.0109, 9.2895) | 9.54E-187 | 9.44E-185 | YES |
| VALPROIC ACID | 5.5164(4.6263, 6.5192) | 5.60E-85 | 5.55E-83 | YES |
| BREXPIPRAZOLE | 22.4297(17.0898, 28.9199) | 2.90E-119 | 2.88E-117 | YES |
| VENLAFAXINE | 5.9154(5.312, 6.5657) | 2.42E-237 | 2.39E-235 | YES |
| FLUOXETINE | 6.3135(5.4372, 7.2843) | 7.39E-135 | 7.32E-133 | YES |
| LEVOFLOXACIN | 7.7752(7.0627, 8.5374) | 0 | 0 | YES |
| CITALOPRAM | 6.6893(5.7853, 7.688) | 1.66E-151 | 1.64E-149 | YES |
| MONTELUKAST | 6.2571(5.5559, 7.0195) | 1.13E-207 | 1.12E-205 | YES |
| TETRABENAZINE | 39.217(31.0474, 48.9809) | 5.38E-219 | 5.32E-217 | YES |
| BACLOFEN | 6.9239(5.4557, 8.6467) | 3.97E-61 | 3.93E-59 | YES |
| ESCITALOPRAM | 5.2932(4.4837, 6.1989) | 1.43E-90 | 1.41E-88 | YES |
| LISDEXAMFETAMINE | 9.4485(8.2626, 10.7534) | 5.06E-245 | 5.01E-243 | YES |
| DEUTETRABENAZINE | 51.2754(36.7789, 70.0541) | 2.11E-127 | 2.09E-125 | YES |
| ATOMOXETINE | 4.6587(4.0069, 5.3824) | 5.90E-93 | 5.85E-91 | YES |
| CARIPRAZINE | 17.6508(12.244, 24.6217) | 9.74E-59 | 9.64E-57 | YES |
| PRAMIPEXOLE | 10.3272(7.8605, 13.292) | 3.13E-68 | 3.10E-66 | YES |
| MIRTAZAPINE | 7.1579(6.1266, 8.3048) | 4.06E-142 | 4.02E-140 | YES |
| ROPINIROLE | 12.7862(9.7868, 16.3855) | 5.39E-84 | 5.34E-82 | YES |
| CLONAZEPAM | 8.4199(7.0644, 9.9487) | 1.20E-131 | 1.18E-129 | YES |
| ISTRADEFYLLINE | 35.1625(5.4922, 127.4777) | 2.83E-06 | 0.000279922 | YES |
| ASENAPINE | 19.4358(14.7959, 25.0675) | 3.09E-108 | 3.06E-106 | YES |
| CARBAMAZEPINE | 3.8003(2.9797, 4.7637) | 5.64E-29 | 5.58E-27 | YES |
| ONDANSETRON | 9.5458(8.0579, 11.2185) | 1.34E-157 | 1.33E-155 | YES |
| OXCARBAZEPINE | 3.8701(2.7677, 5.241) | 7.96E-17 | 7.88E-15 | YES |
| LEVODOPA | 28.0229(17.0588, 43.5206) | 1.06E-44 | 1.05E-42 | YES |
| LORAZEPAM | 6.3389(4.9462, 7.9819) | 7.44E-52 | 7.37E-50 | YES |
| PHENYTOIN | 5.0012(3.982, 6.187) | 1.26E-46 | 1.24E-44 | YES |
| ROTIGOTINE | 11.2011(8.6022, 14.3075) | 1.30E-77 | 1.29E-75 | YES |
| RIVASTIGMINE | 6.2612(4.5858, 8.3162) | 9.36E-34 | 9.27E-32 | YES |
| DONEPEZIL | 8.528(6.6504, 10.746) | 7.43E-69 | 7.35E-67 | YES |
| LITHIUM | 9.5423(7.1086, 12.5081) | 2.00E-55 | 1.98E-53 | YES |
| ENTACAPONE | 33.868(21.9036, 50.2209) | 1.08E-62 | 1.07E-60 | YES |
| AMANTADINE | 26.5764(19.5606, 35.3094) | 1.57E-105 | 1.55E-103 | YES |
| VIGABATRIN | 3.6828(2.4854, 5.2286) | 5.29E-12 | 5.24E-10 | YES |
| MEMANTINE | 11.0391(8.6295, 13.8859) | 1.98E-87 | 1.96E-85 | YES |
| LUMATEPERONE | 17.9829(12.5355, 24.9887) | 6.13E-61 | 6.07E-59 | YES |
| CARBIDOPA;ENTACAPONE;LEVODOPA | 38.1498(25.2285, 55.6003) | 1.73E-73 | 1.71E-71 | YES |
| RASAGILINE | 17.6703(12.8555, 23.6617) | 2.14E-76 | 2.12E-74 | YES |
| PROPOFOL | 4.4569(3.4101, 5.7056) | 4.05E-30 | 4.01E-28 | YES |
| CILASTATIN;IMIPENEM | 10.0825(8.032, 12.4753) | 2.75E-94 | 2.72E-92 | YES |
| PROMETHAZINE | 15.2096(10.9691, 20.5253) | 2.38E-65 | 2.35E-63 | YES |
| CLOBAZAM | 5.6434(3.2826, 8.9793) | 1.15E-11 | 1.13E-09 | YES |
| OPICAPONE | 67.1909(22.0626, 170.0901) | 1.45E-16 | 1.43E-14 | YES |
| VILAZODONE | 6.0618(3.7525, 9.2031) | 2.44E-15 | 2.42E-13 | YES |
